# Extensive Losses of Photosynthesis Genes in the Plastome of a Mycoheterotrophic Orchid, *Cyrtosia septentrionalis* (Vanilloideae: Orchidaceae)

**DOI:** 10.1093/gbe/evz024

**Published:** 2019-02-01

**Authors:** Young-Kee Kim, Sangjin Jo, Se-Hwan Cheon, Min-Jung Joo, Ja-Ram Hong, Myoung Hai Kwak, Ki-Joong Kim

**Affiliations:** 1 Division of Life Sciences, Korea University, Seoul, Korea; 2 Department of Plant Resources, National Institute of Biological Resources, Incheon, Korea

**Keywords:** plastome, gene loss, inverted repeat contraction, gene relocation, *Cyrtosia septentrionalis*, mycoheterotrophy

## Abstract

*Cyrtosia septentrionalis* is an achlorophyllous mycoheterotrophic orchid in the subfamily Vanilloideae (Orchidaceae). This article reports *C. septentrionalis*’s complete plastome sequence and compare it with other orchid plastomes with a same mycoheterotrophic nutritional mode. The *C. septentrionalis* plastome has decreased to 96,859 bp in length, but it still maintains a quadripartite structure. The *C. septentrionalis* plastome contains 38 protein-coding genes, 25 tRNA genes, and four ribosomal RNA genes. Most genes related to photosynthesis have been lost, whereas the majority of housekeeping genes remain; this pattern corresponds to the end of stage 3 gene degradation. The inverted repeat regions of the *C. septentrionalis* plastome have decreased to 10,414 bp and mainly contain the gene *ycf*2. A block consisting of four *rrn* genes and *rps*7 and *rps*12 has shifted to a small single-copy region. As a result, the small single-copy region was found to be expanded, despite the loss of all *ndh* genes in the region. Three inversion mutations are required to explain the *C. septentrionalis* plastome’s current gene order. The species is endangered, and these results have implications for its conservation.

## Introduction

The family Orchidaceae consists of 736 genera, comprising 28,000 species (Christenhusz and Byng 2016), of which the plastome sequences of 116 species from 38 genera have been completely decoded (NCBI database, July 7, 2018). Although most Orchidaceae species have a photosynthetic nutritional mode that is generally similar to other plants, 232 species belonging to 43 genera do not photosynthesize—or they photosynthesize at insignificant levels—and instead rely on mycoheterotrophy for nutrition (Merckx et al. 2013). Among the five subfamilies of Orchidaceae, complete mycoheterotrophism is observed in three subfamilies—Vanilloideae, Orchidoideae, and Epidendroideae—but not in Cypripedioideae or Apostasioideae.

To date, plastome studies on mycoheterotrophic orchids have been performed for 20 species from 10 genera. All of these genera belong to the Epidendroideae subfamily, except for one *Rhizanthella* species (Delannoy et al. 2011), which belongs to Orchidoideae. These include one *Aphyllorchis* species (Feng et al. 2016), one *Cephalanthera* species (Feng et al. 2016), seven *Corallorhiza* species (Barrett and Davis 2012; Barrett et al. 2018), one *Cymbidium* species (Kim et al. 2017; Kim et al. 2018), two *Epipogium* species (Schelkunov et al. 2015), one *Eulophia* species (Huo et al. 2017), one *Gastrodia* species (Yuan et al. 2018), one *Hexalectris* species (Barrett and Kennedy 2018), and four *Neottia* species (Logacheva et al. 2011; Feng et al. 2016). The plastomes of *Platanthera japonica* and *Cremastra appendiculata* have also been decoded (Dong et al. 2018), revealing typical photosynthetic plastomes, even some other congeneric species are mycoheterotrophy.

Plastomes of mycoheterotrophic plants that have lost their photosynthetic ability have been reported to degrade rapidly (Braukmann and Stefanović 2012; Wicke et al. 2013; Wu et al. 2017). Mycoheterotrophic orchids also lost their photosynthetic ability and various levels of gene loss have been observed in the orchid plastomes of 22 species in 10 genera (Logacheva et al. 2011; Barrett and Davis 2012; Schelkunov et al. 2015; Feng et al. 2016; Barrett et al. 2018). With regard to gene loss patterns in these plastomes, *ndh* genes are usually lost first, followed by photosynthetic genes (*atp*, *psa*, *psb*, and *pet* gene classes) and housekeeping genes (*rps*, *rpl*, *trn*, and *rrn* gene classes), in order of precedence (Barrett and Davis 2012; Wicke et al. 2016; Graham et al. 2017).

Of the 14 genera (245 species) in the Vanilloideae subfamily (Chase et al. 2015), five genera (41 species) are classified as mycoheterotrophic orchids (Merckx et al. 2013). However, only plastomes of photosynthetic *Vanilla* species have been reported (Lin et al. 2015; Amiryousefi et al. 2017; Niu et al. 2017), and there is no complete plastome sequence of a mycoheterotrophic vanilloid orchid. Therefore, this study aimed to decode the plastome of *Cyrtosia septentrionalis* (Rchb.f.) Garay, a mycoheterotrophic Vanilloideae.

The tribe Vanilleae of subfamily Vanilloideae consists of 9 genera, 169 species (Chase et al. 2015). The phylogenetic position of *Cyrtosia* in Vanilleae has been well established using various molecular markers such as plastid *psa*B, *rbc*L, and *psb*C (Cameron 2004; Cameron and Carmen Molina 2006), mitochondrial *atp*A and *nad*1B-C (Cameron 2009), and nuclear ribosomal RNA and *xdh* sequences (Cameron 2009; Górniak et al. 2010). These studies suggest that *Cyrtosia*, *Erythrorchis*, and *Pseudovanilla* form a clade that is sister to *Vanilla*. Complete plastome sequence data are available for *Vanilla* but not for the other three genera. Therefore, we selected the *Vanilla* plastome as a reference sequence to compare the structure and gene contents of the *Cyrtosia* plastome for this study.

Five species of *Cyrtosia* are distributed throughout the tropical and subtropical regions of Southeast Asia (Merckx et al. 2013); among them, *C. septentrionalis* is found in warm areas of Japan, China, and South Korea (Lee 2011). *Cyrtosia septentrionalis* is a perennial plant that grows to around 50 cm tall. It is a nonphotosynthetic orchid that has white subterranean rhizomes, red aerial stems, and no leaves. Its flowers bloom in early summer, and its red fruits ripen from summer through autumn (Lee 2011). Its pharmacological value makes the species a target for overcollecting, and as a result it has been designated a legally protected species and is protected by the Korean government under the Biodiversity Conservation Act.

This study completely decoded the plastome of *C. septentrionalis* using next-generation sequencing. These data revealed outstanding cases of gene loss patterns, inverted repeat (IR) contraction and expansion, and gene relocation, and these are discussed in terms of mycoheterotrophy orchid plastomes in general. Furthermore, data on simple sequence repeats (SSRs) with large variability are also presented. These findings offer strategies not only for future plastome studies but also for population genetic studies and efforts to conserve this endangered species.

## Materials and Methods

A living *C. septentrionalis* individual was collected from Jinan-gun, Jeollabuk-do, South Korea, with a collection permit. Genomic DNA was extracted using a G-spinII Plant Genomic DNA extraction kit (iNtRON, Seongnam, Korea). The extracted DNA was deposited in the Plant DNA Bank in Korea under accession number PDBK2016-1045.

Approximately 100 ng of extracted DNA (270.30 ng/µl) was used for library construction and raw sequence reads were generated using Illumina MiSeq (San Diego, CA). For trimming and normalization of raw reads, BBDuk version 37.64 and BBNorm version 37.64—both implemented in Geneious 11.1.2 (Kearse et al. 2012)—were used with kmer length of 27. The trimmed reads were assembled de novo in the Geneious assembler using two different methods. For the first method, plastid reads were filtered and collected from trimmed reads using *Vanilla planifolia* as a reference (GenBank accession number NC036809); the collected plastid reads were then subjected to de novo assembly. The reference-guided assembly method was used to assemble de novo results into complete plastome sequences. For the second method, all redundant reads were removed from trimmed reads by the normalization process. The normalized reads were subjected to de novo assembly and eight plastome contigs were recovered. All redundant reads were mapped to the plastome contigs and finally a single plastome contig was recovered. Possible sequence errors were corrected using redundant reads.

The two de novo assembly methods generated a single, identical contig. The plastome was annotated using the National Center for Biotechnology Information’s (NCBI) BLAST and Geneious 11.1.2 and tRNAscan-SE (Lowe and Chan 2016).

Twenty-nine orchid plastome sequences were downloaded from the NCBI to compare genes (supplementary table S1, Supplementary Material online). Genomic tandem repeats were identified using Phobos v3.3.12 (Mayer 2010). Only perfect repeats with a minimum total length of 10 bp were located. The plastome of *C. septentrionalis* was aligned with two sequences, *V. planifolia* and *Habenariaradiata* (NC035834), using the progressiveMAUVE (Darling et al. 2010) method to detect genomic rearrangement. A circular plastome map was visualized in OGDraw (Lohse et al. 2007).

For phylogenetic tree construction, 79 protein-coding gene and four rRNA gene sequences were aligned using the MUSCLE v.3.8.425 program (Edgar 2004), which was implemented in Geneious 11.1.2. The aligned sequences were 77,315 bp in length. A maximum likelihood analysis was conducted using RAxML v 7.7.1 (Stamatakis et al. 2008) with GTR base substitution model, which was suggested by PAUP modeltest (Posada and Crandall 1998).

## Results and Discussion

**Fig. 1.**
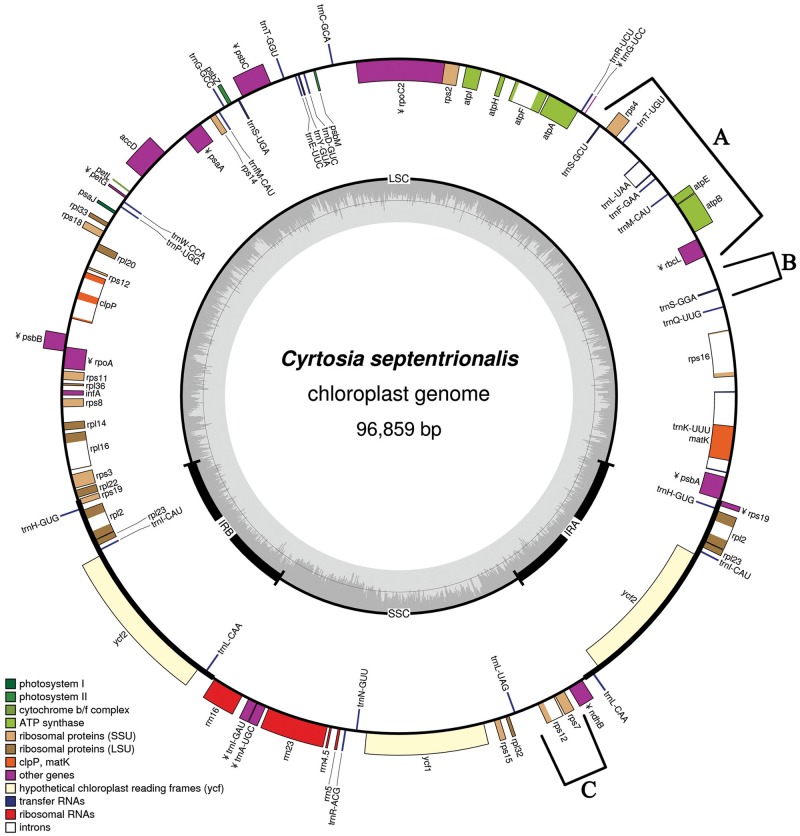
—Plastid circle map of *Cyrtosia septentrionalis*. Pseudogenes are marked with the letter ¥. Three inversion regions along the *C. septentrionalis* plastome are marked on the circle map (A, 7.7-kb inversion; B, 1.4-kb inversion; C, 1.8-kb inversion).

**Fig. 2.**
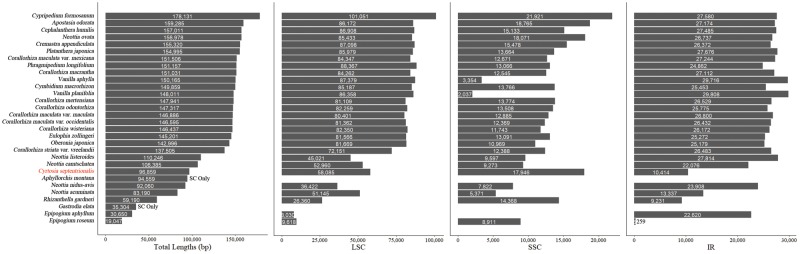
—General features of *Cyrtosia septentrionalis* and 29 other orchid plastomes. All 21 of the decoded mycoheterotrophic orchid plastomes are included except *Hexalectris warnockii*, which was not available in the NCBI database. Species are organized by plastid size.

A total of 12,363,464 trimmed and 1,851,162 normalized reads were recovered from 12,551,898 raw reads, which were an average of 301 bp in length. Of these, 316,705 reads (2.76%) were plastid reads. Average coverage depth was 709 times for each site. The complete plastome of *C. septentrionalis* was found to be 96,859 bp in length (fig. 1). The plastome shows a typical quadripartite structure, with 58,085 bp in large single copy (LSC), 17,946 bp in small single copy (SSC), and 10,414 bp in IR regions (fig. 2 and supplementary table S1, Supplementary Material online). The plastome size of nonphotosynthetic *C. septentrionalis* was 65% that of the photosynthetic *Vanilla planitifolia*, and both belong to tribe Vanilleae.

The *C. septentrionalis* plastome size shows a medium range of variation compared with other nonphotosynthetic orchids (fig. 2). Initially, it seems similar to the plastomes of *Aphyllorchis montana* and *Neottia nidus-avis* (fig. 2), but a detailed comparison revealed quite different characteristics between the three species. Although *C. septentrionalis* and *N. nidus-avis* show quadripartite structures, *A. montana* only contains the SC region, as the IR region has been lost. In addition, the sizes of the LSC, IR, and SSC regions are remarkably different between the plastomes of *C. septentrionalis* and *N. nidus-avis*. When only considering the size of each region, the plastome of *C. septentrionalis* shows features that are not found in any other orchid. This can be explained by the following three evolutionary phenomena, which we discuss below: gene loss, IR boundary shift, and gene relocation.

**Fig. 3.**
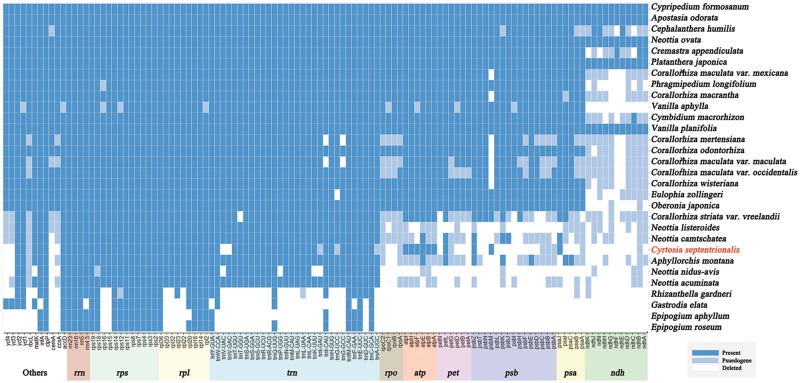
—Summary of gene content for *Cyrtosia septentrionalis* and 29 other orchid plastomes. Blue-colored boxes represent active genes, light blue-colored boxes indicate pseudogenes, and blank boxes denote loss of the gene. Species are organized by plastome size.

The plastomes of photosynthetic land plants usually contain about 113 genes. These include 79 protein-coding genes, 30 tRNA genes, and four ribosomal RNA genes (Shinozaki et al. 1986; Kim and Lee 2004). However, the plastome of *C. septentrionalis* only has 38 protein-coding genes, 25 tRNA genes, and four ribosomal RNA genes (fig. 1 and supplementary table S2, Supplementary Material online). This means that around 52% of the protein-coding genes and 17% of the tRNA genes have been lost or pseudogenized. Most of the lost genes are those involved in photosynthesis. Initially, all 11 *ndh* genes were lost (fig. 3). Of the 26 genes involved in photoelectron transfer (*psa, psb*, and *pet*), only four—*psa*J, *psb*M, *psb*Z, and *pet*L—remain. However, all six genes that form ATP synthase are present in a functional form. Furthermore, *ccs*A*, cem*A*, rbc*L*, ycf*3, and *ycf*4 genes, which are directly or indirectly involved in photosynthesis, were also lost. However, *acc*D*, clp*P*, mat*K*, inf*A*, ycf*1, and *ycf*2, involved in plastid metabolism and housekeeping, are still present. In addition, all 25 genes (*rps, rpl*, and *rrn*) that make ribosomal proteins and ribosomal RNA are present, and 25 out of 30 tRNA genes (*trn*) are present. On the other hand, all four RNA polymerase genes were lost.

In summary, most of *C. septentrionalis*’s plastome genes involved in photosynthesis have been lost, whereas the majority of its housekeeping genes are still present (fig. 3). Gene losses occurred at levels similar to those reported for *Corallorhiza striata* and *Neottia camtschatea* (Barrett and Davis 2012; Feng et al. 2016). Compared with the reported plastome degradation progression stages of nonphotosynthetic plants (Barrett and Davis 2012; Wicke et al. 2016; Graham et al. 2017), the plastome of *C. septentrionalis* is considered to correspond to the end of plastome degradation stage 3. Eight genes have intron(s) in the *C. septentrionalis* plastome: *atp*F, *clp*P, *rps*12, *rps*16, *rpl*16, *rpl*2, *trn*K(UUU), and *trn*L(UAA). Among them, the type IIA intron *trn*K(UUU) is often absent in other nonphotosynthetic plastomes such as *Cuscuta* and *Pilostyles* (McNeal et al. 2009; Graham et al. 2017), but it is present in the *C. septentrionalis* plastome. The same intron is also found in the various orchid plastomes, such as *A.montana*, *Corallorhiza striata* var. *vreelandii*, *Neottia acuminata*, *N. camtschatea*, and *Neottialisteroides* (Barrett and Davis 2012; Feng et al. 2016).

The plastome of *C. septentrionalis* has a general quadripartite structure because it contains an IR region. However, the IR region has been drastically reduced to 10,414 bp, most of which is occupied by *ycf*2, *rpl*23, *rpl*2, and three *trn* genes (figs. 1 and 2). This is markedly different from most common land plant plastomes and is even significantly different from plastomes of plants from the same family (Orchidaceae). The IR region usually contains four *rrn* genes, *rps*7, *rps*12, three to five *trn* genes, and *ycf*2. However, in the *C. septentrionalis* plastome, all of those genes are located in the SSC region. When all *ndh* genes located in the SSC region have been lost, the SSC region is usually shortened (Lin et al. 2015; Feng et al. 2016). However, *C. septentrionalis* lost all of its *ndh* genes, but the SSC region had actually extended to 17,946 bp whereas the IR region was greatly shortened. This phenomenon is related to the fact that IR contraction occurred at the SSC boundary. This can be explained as the result of gene loss and IR expansion/contraction. The IR region is usually maintained, even in the case of mycoheterotrophic orchids in which various plastid gene losses have occurred. This can be attributed to the fact that, although photosynthetic function has been lost, the plastid’s basic function remains (Schelkunov et al. 2015; Feng et al. 2016; Barrett et al. 2018). The presence of the IR is believed to contribute to the plastome’s stability (Palmer and Thompson 1982). In nonphotosynthetic orchids, the IR has only been reported to be lost in the plastomes of *Gastrodia elata* and *Aphylloorchis montana* (Feng et al. 2016; Yuan et al. 2018).

**Fig. 4.**
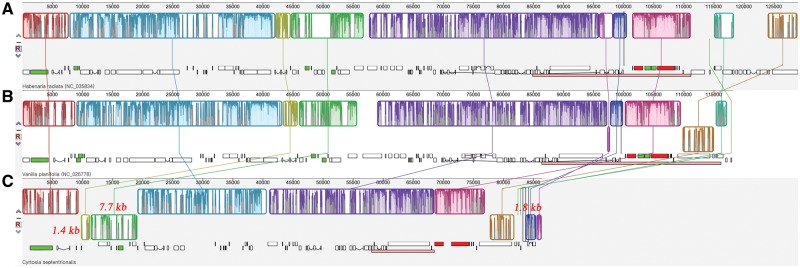
—Plastome alignment using progressive MAUVE including (*A*) *Habenaria radiata* (NC035834), (*B*) *Vanilla planifolia* (NC026778), and (*C*) *Cyrtosia septentrionalis* sequences. Inversions between *C*. *septentrionalis* and *V*. *planifolia* are indicated as a red letter with its size. *Vanilla* and *Cyrtosia* are phylogenetically closely related genera in the tribe Vanilleae, but are not sister group (Cameron 2004, 2009; Cameron and Carmen Molina 2006; Górniak et al. 2010).

To explain why the gene order of the *C. septentrionalis* plastome differs from that of *V.planifolia*, even though they belong to the same tribe (supplementary fig. S1, Supplementary Material online), three inversions must be assumed (fig. 4). Based on the results of comparative studies, it can be inferred that two inversions occurred in the LSC region and one in the SSC region. The 7.7-kb inversion in the LSC region includes the pseudo *rbc*L–*atp*B–*atp*E–*rps*4 gene block, which is located alongside another 1.4-kb inversion in the *ycf*3 and *trn*S-GGA gene region. The third inversion is 1.8 kb long, is found in the SSC region, and includes the *rps*7 and *rps*12 genes (fig. 4). The three inversions found in the *C. septentrionalis* plastome are unique to this species; however, they are not special in terms of nonphotosynthetic plants, as gene losses are usually accompanied by gene relocations, for example, the *atp*F–*atp*H region inversion in *A.montana* (Feng et al. 2016), 11-kb inversion between *psb*A–*rps*2 in *N.acuminata* (Feng et al. 2016), 16-kb inversion between *pet*B–*cem*A in *Corallorhiza maculata* (Barrett et al. 2014), and 29-kb inversion between *ycf*3–*trn*S-GCU in *Hexalectris warnockii* (Barrett and Kennedy 2018).

A large number of SSRs are present in the *C. septentrionalis* plastome. Of the 135 SSRs, the majority (85) are pentanucleotide repeats, followed by 26 mononucleotide, 15 dinucleotide, seven tetranucleotide, and two trinucleotide repeats (supplementary table S3, Supplementary Material online). In nonphotosynthetic orchid plastomes, phenomena such as gene loss, IR contraction, and gene relocation seem to occur in a complex manner during evolutionary processes. These processes are considered to be relaxation processes necessary to facilitate gene exclusion while maintaining the function of the remaining genes.

## Supplementary Material

Supplementary data are available at *Genome Biology and Evolution* online.

## Supplementary Material

Supplementary Data

## Footnotes

**Data deposition:** Data have been deposited at NCBI GenBank under the accession MH615835.
