# Supplementary material for: Extensive Losses of Photosynthesis Genes in the Plastome of a Mycoheterotrophic Orchid, Cyrtosia septentrionalis (Vanilloideae: Orchidaceae)
Source: Genome Biol Evol. 2019 Feb 1;11(2):565–71. doi: 10.1093/gbe/evz024 (PMC6390903; doi:10.1093/gbe/evz024)
Supplement: Supplementary Data [file evz024_supp.zip › Supplemantal Data.docx]

**Supplemantal Data**

**Supplementary table S1.** General information about the plastome sequences used in this study.

**Supplementary table S2.** Gene contents of the *Cyrtosia septentrionalis* plastome.

**Supplementary table S3.** List of simple sequence repeats (SSRs) along the *Cyrtosia septentrionalis* plastome.

**Supplementary figure S1.** A maximum likelihood tree inferred from 79 protein coding and four rRNA genes for the 30 orchid species used in Fig. 3.
