# Supplementary material for: Extensive Losses of Photosynthesis Genes in the Plastome of a Mycoheterotrophic Orchid, Cyrtosia septentrionalis (Vanilloideae: Orchidaceae)
Source: Genome Biol Evol. 2019 Feb 1;11(2):565–71. doi: 10.1093/gbe/evz024 (PMC6390903; doi:10.1093/gbe/evz024)

**Supplementary table S1.** General information about the plastome sequences used in this study.

| Species | NCBI# | Lengths | LSC | SSC | IR |
|---|---|---|---|---|---|
| *Epipogium roseum* | NC_026448.1 | 19,047 | - | - | - |
| *Epipogium aphyllum* | NC_026449.1 | 30,650 | - | - | - |
| *Gastrodia elata* | NC_037409.1 | 35,304 | - | - | - |
| *Rhizanthella gardneri* | NC_014874.1 | 59,190 | 26,360 | 14,368 | 9,231 |
| *Neottia acuminata* | NC_030709.1 | 83,190 | 51,145 | 5,371 | 13,337 |
| *Neottia nidus-avis* | NC_016471.1 | 92,060 | 36,422 | 7,822 | 23,908 |
| *Aphyllorchis montana* | NC_030703.1 | 94,559 | - | - | - |
| *Cyrtosia septentrionalis* | MH615835 | 96,859 | 58,085 | 17,946 | 10,414 |
| *Neottia camtschatea* | NC_030707.1 | 106,385 | 52,960 | 9,273 | 22,076 |
| *Neottia listeroides* | NC_030713.1 | 110,246 | 45,021 | 9,597 | 27,814 |
| *Corallorhiza striata* var. *vreelandii* | JX087681 | 137,505 | 72,151 | 12,388 | 26,483 |
| *Oberonia japonica* | NC_035832.1 | 142,996 | 81,669 | 10,969 | 25,179 |
| *Eulophia zollingeri* | NC_037212 | 145,201 | 81,566 | 13,091 | 25,272 |
| *Corallorhiza wisteriana* | NC_025663 | 146,437 | 82,350 | 11,743 | 26,172 |
| *Corallorhiza maculata* var. *occidentalis* | KM390016 | 146,595 | 81,362 | 12,369 | 26,432 |
| *Corallorhiza maculata* var. *maculata* | KM390014 | 146,886 | 80,401 | 12,885 | 26,800 |
| *Corallorhiza odontorhiza* | NC_025664 | 147,317 | 82,259 | 13,508 | 25,775 |
| *Corallorhiza mertensiana* | NC025661 | 147,941 | 81,109 | 13,774 | 26,529 |
| *Vanilla planifolia* | NC_026778.1 | 148,011 | 86,358 | 2,037 | 29,808 |
| *Cymbidium macrorhizon* | KY354040 | 149,859 | 85,187 | 13,766 | 25,453 |
| *Vanilla aphylla* | NC_035320.1 | 150,165 | 87,379 | 3,354 | 29,716 |
| *Corallorhiza macrantha* | NC_025660 | 151,031 | 84,262 | 12,545 | 27,112 |
| *Phragmipedium longifolium* | NC_028149.1 | 151,157 | 88,367 | 13,066 | 24,862 |
| *Corallorhiza maculata* var. *mexicana* | KM390015 | 151,506 | 84,347 | 12,671 | 27,244 |
| *Platanthera japonica* | NC_037440 | 154,995 | 85,979 | 13,664 | 27,676 |
| *Cremastra appendiculata* | NC_037439 | 155,320 | 87,098 | 15,478 | 26,372 |
| *Neottia ovata* | NC_030712.1 | 156,978 | 85,433 | 18,071 | 26,737 |
| *Cephalanthera humilis* | NC_030706 | 157,011 | 86,908 | 15,133 | 27,485 |
| *Apostasia odorata* | NC_030722.1 | 159,285 | 86,172 | 18,765 | 27,174 |
| *Cypripedium formosanum* | NC_026772.1 | 178,131 | 101,051 | 21,921 | 27,580 |

**Supplementary table S2.** Gene contents of the *Cyrtosia septentrionalis* plastome.

| Category for genes | Group of genes | Genes |
|---|---|---|
| Self replication | rRNA genes | *rrn*16, *rrn*23, *rrn*4.5, *rrn*5 |
| | tRNA genes | 25 *trn* genes(3 in IR regions with asterisk) *trn*C-GCA, *trn*D-GUC, *trn*E-UUC, *trn*F-GAA, *trn*fM-CAU, *trn*G-GCC, *trn*H-GUG*, *trn*I-CAU*, *trn*K-UUU, *trn*L-CAA*, *trn*L-UAA, *trn*L-UAG, *trn*M-CAU, *trn*N-GUU, *trn*P-UGG, *trn*Q-UUG, *trn*R-ACG, *trn*R-UCU, *trn*S-GCU, *trn*S-GGA, *trn*S-UGA, *trn*T-GGU, *trn*T-UGU, *trn*W-CCA, *trn*Y-GUA |
| | Small subunit of ribosome | *rps*2, *rps*3, *rps*4, *rps*7, *rps*8, *rps*11, *rps*12, *rps*14, *rps*15, *rps*16, *rps*18, *rps*19 |
| | Large subunit of ribosome | *rpl*2(x2), *rpl*14, *rpl*16, *rpl*20, *rpl*22, *rpl*23(x2), *rpl*32, *rpl*33, *rpl*36 |
| Photosynthesis related | Subunits of photosystem 1 | *psa*J |
| | Subunits of photosystem 2 | *psb*M, *psb*Z |
| | Subunits of cytochrome b/f complex | *pet*L |
| | Subunits of ATP synthase | *atp*A, *atp*B, *atp*E, *atp*F, *atp*H, *atp*I |
| Other genes | Translational initiation factor | *inf*A |
| | Maturase | *mat*K |
| | Protease | *clp*P |
| | Subunit of Acetyl-CoA-carboxylase | *acc*D |
| Genes of unknown functions Open Reading Frames (ORF, *ycf*1) | | *ycf*1, *ycf*2(x2) |

**Supplementary table S3.** List of simple sequence repeats (SSRs) along the *Cyrtosia septentrionalis* plastome.

| Type | Sequence | Length | Repeating Unit | Region | Position |
|---|---|---|---|---|---|
| Mono | TTTTTTTTTT | 10 | T | LSC | IGS |
| Mono | AAAAAAAAAA | 10 | A | LSC | IGS |
| Mono | AAAAAAAAAA | 10 | A | LSC | IGS |
| Mono | AAAAAAAAAAA | 11 | A | LSC | IGS |
| Mono | AAAAAAAAAA | 10 | A | LSC | IGS |
| Mono | AAAAAAAAAA | 10 | A | LSC | IGS |
| Mono | TTTTTTTTTTT | 11 | T | LSC | IGS |
| Mono | AAAAAAAAAAAAAA | 14 | A | LSC | Intron |
| Mono | AAAAAAAAAAA | 11 | A | LSC | IGS |
| Mono | GGGGGGGGGGG | 11 | G | LSC | IGS |
| Mono | TTTTTTTTTT | 10 | T | LSC | IGS |
| Mono | AAAAAAAAAA | 10 | A | LSC | IGS |
| Mono | AAAAAAAAAAAA | 12 | A | LSC | IGS |
| Mono | TTTTTTTTTT | 10 | T | LSC | IGS |
| Mono | AAAAAAAAAAAA | 12 | A | LSC | IGS |
| Mono | TTTTTTTTTT | 10 | T | LSC | IGS |
| Mono | AAAAAAAAAA | 10 | A | LSC | Intron |
| Mono | TTTTTTTTTT | 10 | T | LSC | IGS |
| Mono | TTTTTTTTTTT | 11 | T | LSC | IGS |
| Mono | AAAAAAAAAA | 10 | A | LSC | IGS |
| Mono | TTTTTTTTTTTTTTT | 15 | T | LSC | Intron |
| Mono | GGGGGGGGGGGG | 12 | G | SSC | IGS |
| Mono | AAAAAAAAAA | 10 | A | SSC | CDS |
| Mono | AAAAAAAAAA | 10 | A | SSC | CDS |
| Mono | AAAAAAAAAA | 10 | A | SSC | IGS |
| Mono | AAAAAAAAAA | 10 | A | SSC | CDS |
| Di | TATATATATA | 10 | TA | LSC | Intron |
| Di | ATATATATATATAT | 14 | AT | LSC | Intron |
| Di | TATATATATA | 10 | TA | LSC | Intron |
| Di | ATATATATAT | 10 | AT | LSC | IGS |
| Di | TATATATATATA | 12 | TA | LSC | IGS |
| Di | TATATATATATA | 12 | TA | LSC | Intron |
| Di | ATATATATAT | 10 | AT | LSC | IGS |
| Di | ATATATATAT | 10 | AT | LSC | IGS |
| Di | TATATATATATATATA | 16 | TA | LSC | IGS |
| Di | ATATATATATAT | 12 | AT | LSC | IGS |

| | | | | | |
|---|---|---|---|---|---|
| Di | ATATATATATATATATAT | 18 | AT | LSC | IGS |
| Di | ATATATATATATATAT | 16 | AT | LSC | IGS |
| Di | AGAGAGAGAG | 10 | AG | SSC | CDS |
| Di | ATATATATATAT | 12 | AT | SSC | IGS |
| Di | TATATATATATATA | 14 | TA | SSC | IGS |
| Tri | TATTATTATTAT | 12 | TAT | SSC | IGS |
| Tri | AGAAGAAGAAGA | 12 | AGA | SSC | IGS |
| Tetra | TATTTATTTATT | 12 | TATT | LSC | Intron |
| Tetra | AAATAAATAAAT | 12 | AAAT | LSC | IGS |
| Tetra | GTCTGTCTGTCT | 12 | GTCT | LSC | CDS |
| Tetra | ATAAATAAATAA | 12 | ATAA | LSC | CDS |
| Tetra | ATAGATAGATAG | 12 | ATAG | LSC | Intron |
| Tetra | CAATCAATCAAT | 12 | CAAT | IR | CDS |
| Tetra | ATTCATTCATTC | 12 | ATTC | SSC | IGS |
| Penta | TATATTATAT | 10 | TATAT | LSC | Intron |
| Penta | TAGGGTAGGG | 10 | TAGGG | LSC | CDS |
| Penta | ATAAAATAAA | 10 | ATAAA | LSC | Intron |
| Penta | TATTTTATTT | 10 | TATTT | LSC | Intron |
| Penta | TTTTATTTTA | 10 | TTTTA | LSC | Intron |
| Penta | AATATAATAT | 10 | AATAT | LSC | IGS |
| Penta | ATATAATATA | 10 | ATATA | LSC | IGS |
| Penta | TTATTTTATT | 10 | TTATT | LSC | Intron |
| Penta | AATACAATAC | 10 | AATAC | LSC | Intron |
| Penta | AATAGAATAG | 10 | AATAG | LSC | IGS |
| Penta | TATAATATAA | 10 | TATAA | LSC | IGS |
| Penta | TAATATAATA | 10 | TAATA | LSC | IGS |
| Penta | ATCCAATCCA | 10 | ATCCA | LSC | IGS |
| Penta | ATTTTATTTT | 10 | ATTTT | LSC | IGS |
| Penta | TCAACTCAAC | 10 | TCAAC | LSC | IGS |
| Penta | AACTAAACTA | 10 | AACTA | LSC | IGS |
| Penta | TTTCGTTTCG | 10 | TTTCG | LSC | CDS |
| Penta | TAGATTAGAT | 10 | TAGAT | LSC | IGS |
| Penta | TATACTATAC | 10 | TATAC | LSC | IGS |
| Penta | TTTTATTTTA | 10 | TTTTA | LSC | Intron |
| Penta | TGAATTGAAT | 10 | TGAAT | LSC | Intron |
| Penta | TTTTATTTTA | 10 | TTTTA | LSC | Intron |
| Penta | CATTCCATTC | 10 | CATTC | LSC | IGS |
| Penta | TTTGCTTTGC | 10 | TTTGC | LSC | IGS |
| Penta | TGATTTGATT | 10 | TGATT | LSC | IGS |
| Penta | ATAAGATAAG | 10 | ATAAG | LSC | IGS |

| | | | | | |
|---|---|---|---|---|---|
| Penta | TTCTTTTCTT | 10 | TTCTT | LSC | IGS |
| Penta | CAATACAATA | 10 | CAATA | LSC | IGS |
| Penta | TTTCCTTTCC | 10 | TTTCC | LSC | IGS |
| Penta | TTCTTTTCTT | 10 | TTCTT | LSC | IGS |
| Penta | AATAAAATAA | 10 | AATAA | LSC | IGS |
| Penta | TATTCTATTC | 10 | TATTC | LSC | IGS |
| Penta | TAGGCTAGGC | 10 | TAGGC | LSC | IGS |
| Penta | AAAAGAAAAG | 10 | AAAAG | LSC | IGS |
| Penta | AAATAAAATA | 10 | AAATA | LSC | IGS |
| Penta | TAAGATAAGA | 10 | TAAGA | LSC | IGS |
| Penta | TTGTATTGTA | 10 | TTGTA | LSC | IGS |
| Penta | AGATAAGATA | 10 | AGATA | LSC | IGS |
| Penta | AGCATAGCAT | 10 | AGCAT | LSC | IGS |
| Penta | ACATAACATA | 10 | ACATA | LSC | IGS |
| Penta | TCCTTTCCTT | 10 | TCCTT | LSC | IGS |
| Penta | TGGATTGGAT | 10 | TGGAT | LSC | CDS |
| Penta | ATTTGATTTG | 10 | ATTTG | LSC | IGS |
| Penta | TTTTCTTTTC | 10 | TTTTC | LSC | IGS |
| Penta | TTATATTATA | 10 | TTATA | LSC | IGS |
| Penta | AAAATAAAAT | 10 | AAAAT | LSC | IGS |
| Penta | ATATTATATT | 10 | ATATT | LSC | IGS |
| Penta | TTAAATTAAA | 10 | TTAAA | LSC | IGS |
| Penta | ATTCAATTCA | 10 | ATTCA | LSC | CDS |
| Penta | ATCGAATCGA | 10 | ATCGA | LSC | CDS |
| Penta | AACTAAACTA | 10 | AACTA | LSC | IGS |
| Penta | ATAATATAAT | 10 | ATAAT | LSC | IGS |
| Penta | AATGAAATGA | 10 | AATGA | LSC | IGS |
| Penta | TTTTCTTTTC | 10 | TTTTC | LSC | IGS |
| Penta | AACCTAACCT | 10 | AACCT | LSC | IGS |
| Penta | TTTCTTTTCT | 10 | TTTCT | LSC | IGS |
| Penta | TTCTTTTCTT | 10 | TTCTT | LSC | IGS |
| Penta | TTTCATTTCA | 10 | TTTCA | LSC | IGS |
| Penta | TGATTTGATT | 10 | TGATT | LSC | IGS |
| Penta | AAAGAAAAGA | 10 | AAAGA | LSC | Intron |
| Penta | TTTCTTTTCT | 10 | TTTCT | LSC | IGS |
| Penta | AAACGAAACG | 10 | AAACG | LSC | IGS |
| Penta | TAATCTAATC | 10 | TAATC | LSC | CDS |
| Penta | TCATATCATA | 10 | TCATA | LSC | IGS |
| Penta | AATATAATAT | 10 | AATAT | LSC | IGS |
| Penta | TTTTATTTTA | 10 | TTTTA | LSC | IGS |

| | | | | | |
|---|---|---|---|---|---|
| Penta | TTCTATTCTA | 10 | TTCTA | LSC | Intron |
| Penta | AAATAAAATA | 10 | AAATA | LSC | Intron |
| Penta | ATAAAATAAA | 10 | ATAAA | LSC | Intron |
| Penta | TATTCTATTC | 10 | TATTC | LSC | IGS |
| Penta | TGGATTGGAT | 10 | TGGAT | IR | CDS |
| Penta | ATACCATACC | 10 | ATACC | IR | IGS |
| Penta | GATCCGATCC | 10 | GATCC | IR | CDS |
| Penta | AAGATAAGAT | 10 | AAGAT | IR | IGS |
| Penta | TATCTTATCT | 10 | TATCT | IR | IGS |
| Penta | CTGGTCTGGT | 10 | CTGGT | IR | IGS |
| Penta | ACATAACATA | 10 | ACATA | SSC | IGS |
| Penta | AATGAAATGA | 10 | AATGA | SSC | CDS |
| Penta | GGGAAGGGAA | 10 | GGGAA | SSC | CDS |
| Penta | AGAAAAGAAA | 10 | AGAAA | SSC | CDS |
| Penta | TCCAATCCAA | 10 | TCCAA | SSC | CDS |
| Penta | ATTCCATTCC | 10 | ATTCC | SSC | CDS |
| Penta | TATAGTATAG | 10 | TATAG | SSC | IGS |
| Penta | AGTATAGTAT | 10 | AGTAT | SSC | IGS |
| Penta | ATGCAATGCA | 10 | ATGCA | SSC | IGS |

**Supplementary figure S1.** A maximum likelihood tree inferred from 79 protein coding and four rRNA genes for the 30 orchid species used in Fig. 3.

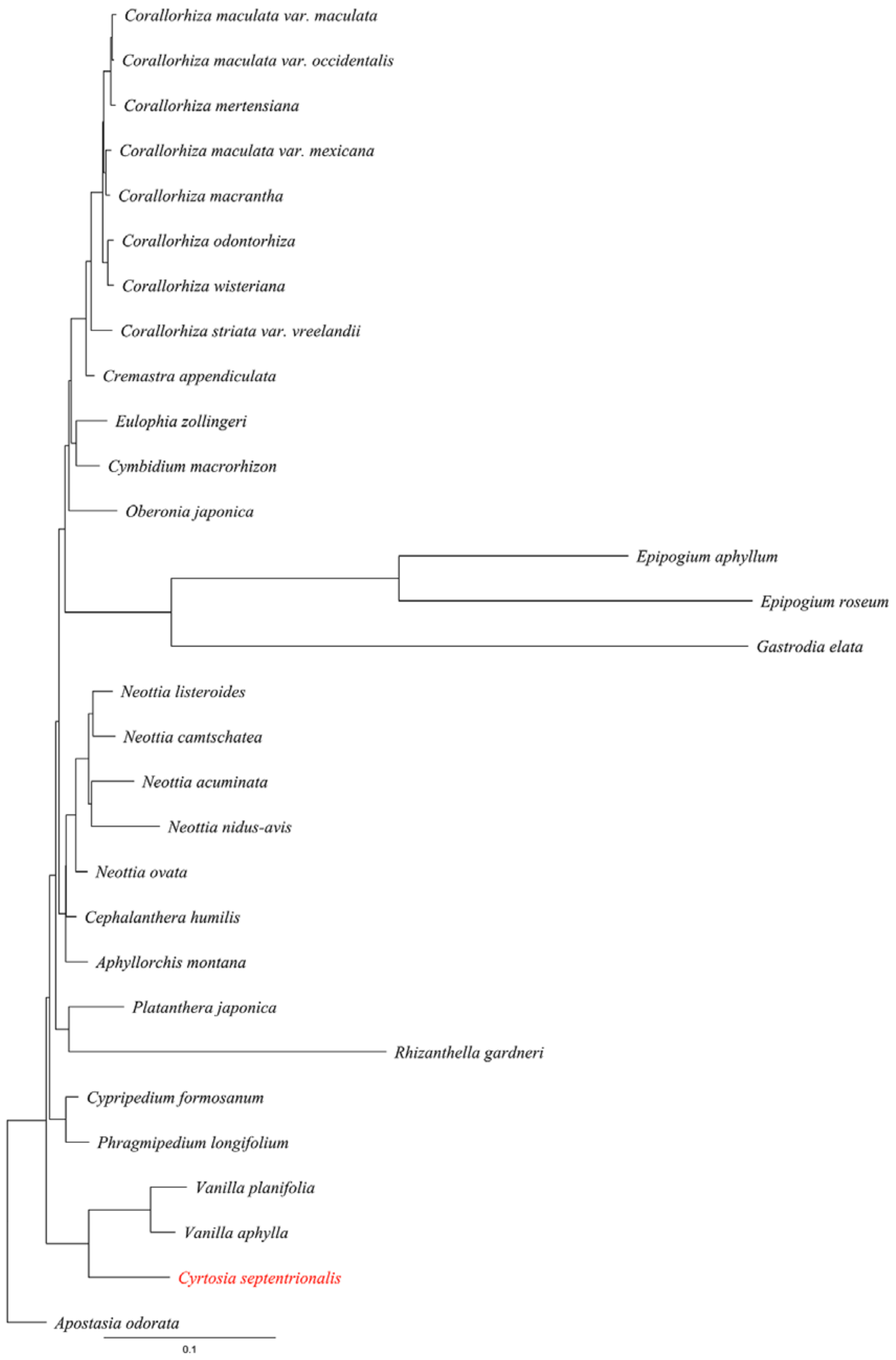

Supplement: Supplementary Data [file evz024_supp.zip › Supplementary data.pdf]
